# Evaluation of Antioxidant Stability of Arbutin and *Pyrus boissieriana Buhse* Leaf Extract

**Published:** 2013

**Authors:** Asieh Khalilpour, Mahdi Pouramir, Fariba Asgharpour

**Affiliations:** 1 *Paramedical Faculty, Babol University of Medical Sciences, Babol, Iran.*; 2 *Cellular and Molecular Biology Research Center (CMBRC), Babol University of Medical Sciences, Babol, Iran.*; 3 *Department of Clinical Biochemistry, Babol University of Medical Sciences, Babol, Iran.*

**Keywords:** Arbutin, *pyrus biossieriana buhse*, antioxidants, lipid oxidation, FRAP assay, TBARS, peroxidase activity

## Abstract

With regard to the importance of antioxidants in foods, cosmetics and pharmaceutics, there are several studies on natural resources for finding rich sources of antioxidants and their role in protecting the body against oxidative stress injuries. The purpose of this study was to investigate the antioxidant stability of arbutin and the *Pyrus boissieriana buhse *Leaf extract and their effects on lipid oxidation in different conditions of temperature and time. Arbutin and the *Pyrus boissieriana buhse *Leaf extract were stored for 14 days in the different conditions of temperature including room, refrigerator and freezer. Total phenolic compounds were measured by the folin-ciocaltea method. Flavonoid compounds were evaluated by aluminum chloride method. Their total antioxidant activity was measured by FRAP (ferric reducing antioxidant power) method and their protection effect on lipid oxidation was measured by TBARS (thiobarbituric acid reactant substances) method. Also, the amount of sustainability for peroxide activities was measured by TMB (Tetra Methyl Benzedrine) method. Polyphenol formed 1.96 mg/g dry weight of *Pyrus boissieriana buhse *Leaf extract and the amount of flavonoid complex was 0.125 mg/g dry weight of *Pyrus boissieriana buhse *Leaf extract. The amount of FRAP was decreased by increasing temperature and time. The amount of lipid oxidation had increased in all samples with time (0**-**14). The stability of peroxide activities decreased in the different conditions of temperature and time. The results of this study show the existence of antioxidant activities with higher stability in storage time and the protective effect of arbutin and *Pyrus boissieriana buhse *Leaf extract on lipid oxidation. Therefore, using arbutin and *Pyrus boissieriana buhse *Leaf extract as a natural resource of antioxidant is suggested for substituting synthetic antioxidants.

Free radicals are atoms or groups of atoms with an odd (unpaired) number of electrons and can be formed when oxygen interacts with certain molecules (1). In fact, free radicals with high energy are very unsustainable. They interact with special chemicals of body and therefore may interfere with natural functions of cells (2). Normally, the defensive system of the body neutralizes these free radicals produced by environmental destructive elements including air pollution, smoking, exposure to sunlight, imperfect breakdown of fat or proteins in the body upon ingestion of cancerigeneous foods such as nitrate, environmental hormones, lead and harmful chemicals, toxic compounds, bacteria and viruses (3). The best way for contrasting with free radicals is using sufficient antioxidants. The anti-oxidant will prevent oxidation of material and also production of free radicals in the body. In natural conditions, there is a balance between the production of free radicals and Reactive oxygen species (ROS) and the power of antioxidant defective system. Although higher production of free radicals or decreasing antioxidant defection increases the condition for free radical damage called oxidative stress (4).

Wild pear with scientific name of Pyrus boissieriana buhse, is native to north of Iran. Leaf and plant's skin in Pyrus kind have a phenol glycoside called arbutin. Arbutin can be found in high amount in some plants including Rosaceae species. It has antioxidant and sunscreen effects and could be used as whitening - formulations of locally used drugs for protection against ultraviolet (5). With regard to the important antioxidant properties of arbutin and *Pyrus boissieriana buhse *Leaf extract and their application in medicine and pharmacy, there are no considerable scientific and laboratorial studies for investigating their antioxi-dant stability. This study was performed to investigate the antioxidant stability of arbutin and *Pyrus boissieriana buhse *Leaf extract and their effect on lipid oxidation under the different conditions of temperature and time.

## Materials and Methods

**Plant material**

*Pyrus boissieriana buhse *Leaf were collected in Babol (north of Iran) in May 2012. The plant material was identified and authenticated by the Mazandran Department of Agricultural Sciences & Natural Resources.

**Extraction**

The fresh Leaf were chopped into small pieces, dried in the shade and then grinded using a blender for extraction. A portion of the material (500 g) was extracted with methanol (63%) for 36 h at room temperature and evaporated to dryness under reduced pressure (6). The extract was stored at **-**20 °C.

**Determination of total phenols**

Folin-Ciocalteu reagent adapted from McDonald (7) was used for the determination of total phenols. Diluted extract (0.5 ml of 1:10, v/v) and phenolic standard were mixed with Folin ciocalteu reagent (5 ml, 1:10 diluted) and aqueous Na_2_CO_3_ (4 ml, 1M). The solutions were heated in a water bath at 45°C for 15 min and the total phenols were determined by spectrophotometry at 765 nm. The standard curve was prepared using 0, 50, 100, 150, 200, 250 mg/l solutions of gallic acid in methanol: water (50:50,v/v). Total phenol values were expressed as gallic acid equivalents (mg/g dry weight) which is a common reference compound. 

**Total flavonoid content**

0.5 ml plant extract was added to 1.5 ml methanol, 0.1 ml aluminum chloride (1%), 0.1 ml potassium acetate (1 M) and 2.8 ml distilled water. After 30 min incubation at room temperature, sample absorbance was determined at 415 nm. Calibration curve of quercetin was prepared in the range of 0-50 mg/ ml in methanol. The results were reported as querceting equivalent, mg/g dry weight (8).

**Total antioxidant activity assay**

Total antioxidant activity was estimated by a FRAP (ferric reducing antioxidant power) assay (9). Briefly, the FRAP reagent contained 2.5 ml of a 10 mmol/L TPTZ (2,4,6-tripyridyl-s-triazine; Sigma) solution in 40 mmol/L HCl plus 2.5 ml of 20 mmol/L FeCl3 and 25 ml of 0.3 mol/L acetate buffer (pH 3.6); the reagent was freshly prepared and warmed at 37 °C. The working FRAP reagent (1.5 ml) was mixed with 50 μl serum or standard in a test tube. After 10 min at 37 °C, the absorbance was determined at 593 nm. FeSO4 at a concentration of 1 mmol/L was used as the standard solution. The final result was expressed as the concentration of antioxidant with a ferric reducing ability equivalent to that of 1 mmol/L FeSO4 (10).

**TBARS test and** **peroxidase activity**

This method is explained by Ahn et al (11). For providing TBARS (thiobarbituric acid reactant substances) solution, TBA/TCA solution and BHT (butyl hydroxy toluene (0.01 /ethanol) are provided. Antioxidant activities were measured in meat with a slight modification (12). Homogenized fish meat (4 ml) was added to all test and extract tubes and arbutin as antioxidant was also added to negative control of distilled water and to the positive control of BHT tubes. All tubes were placed from zero to fourteen days at room temperature, refrigerator (4 ^o^C) and freezer (-20^ o^C). After 3, 7 and 14 days, 4 ml of TBA /TCA and 50 µl of BHT were added to all tubes and placed in boiling bath for 15 minutes. Tubes were centrifuged for 15 minutes at 3000 rpm and the absorption of surfactant was red at 532 nm by spectrophotometer.

Peroxidase activity was measured by using tetra methyl benzidine (TMB). In this study, 20 µl of surfactant was added to 100 µl TMB and H2O2 50:50. Then it was held for 30 min at room temperature under darkness conditions. Color devel-opment was stopped by the introduction of 100 µl of 0.6 N sulfuric acid. The optical density of each well was determined by ELISA reader at 450 nm (13).

**Statistical analysis**

All measurements were repeated 3 times and all data were based on Mean± SD. Variance analysis was done by ANOVA for mean comparison and the results were considered as significant with P<0/05. 

## Results

Extract characteristics: the yield of extraction was 21.6%.

Polyphenol amount was 46 mg which was equal to Gallic acid in 1000 ml *Pyrus boissieriana buhse *Leaf extract. 

Flavonoid concentration was based on querceting 80 μg/ml of methanol extract which was equal to 0.125 mg/g dry weight in *Pyrus boissieriana buhse *Leaf. 

**Fig 1:**
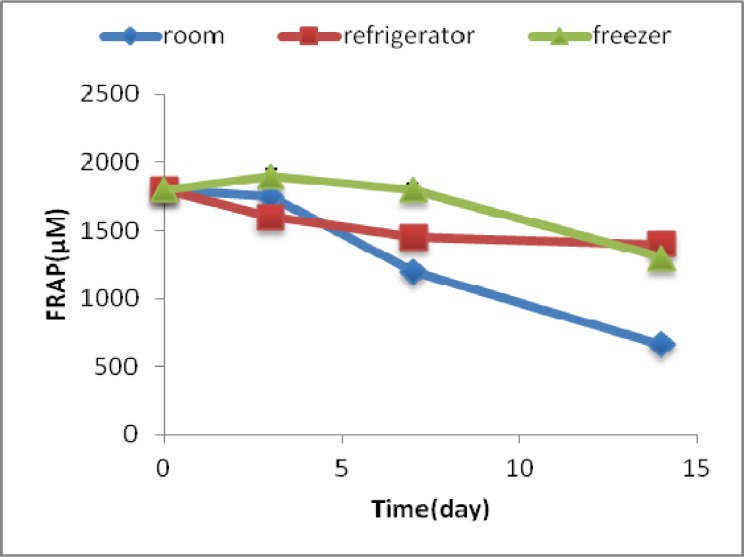
FRAP amount (μ M) of arbutin during time and under different storage conditions

FRAP amount of arbutin in zero time was 1800 ± 40 µM. FRAP amount in each set of samples in different temperatures -room, refri-gerator and freezer- decreased with time. The most important decrease was 63% on 14th day at room temperature (Fig. 1). 

**Fig 2:**
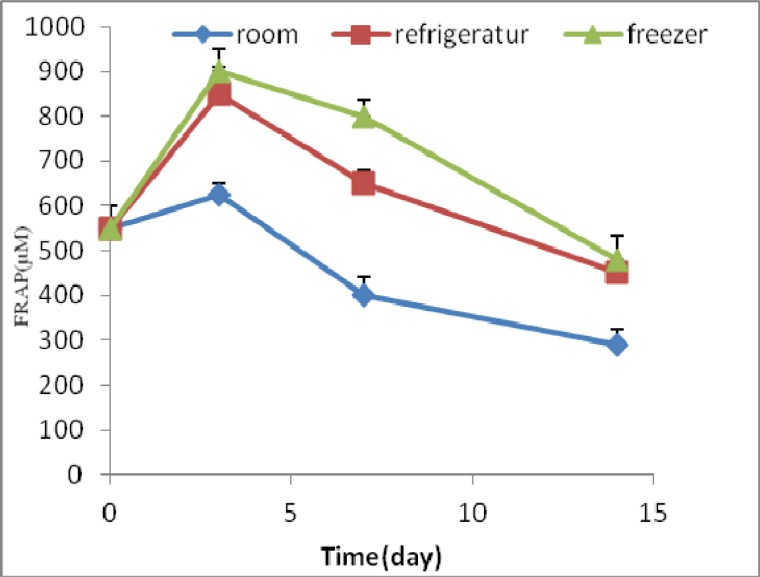
FRAP amount (μ M) of extract during time and under different storage conditions

**Fig 3:**
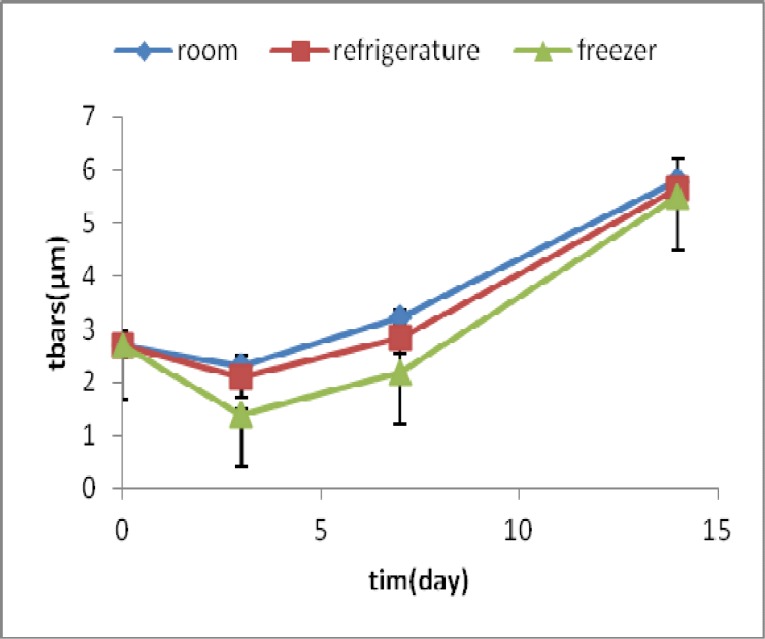
TBARS amounts (μ M) in samples of homogenized fish meat containing arbutin during time and under different storage conditions

**Fig 4:**
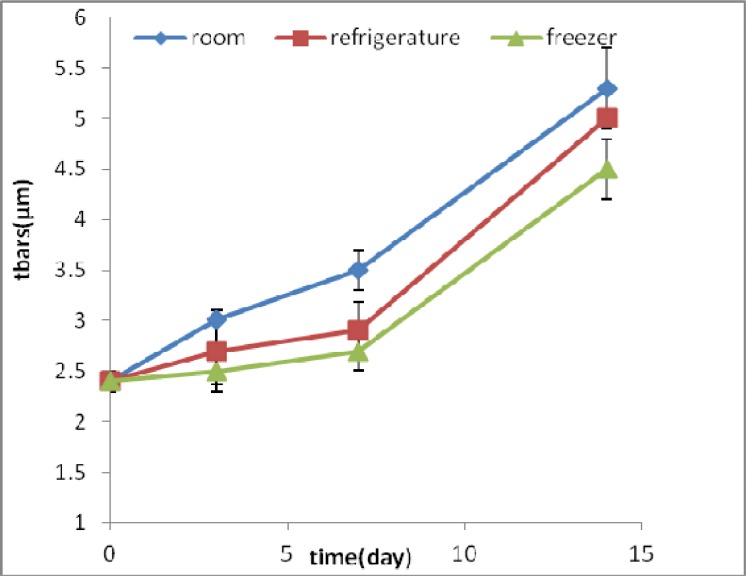
TBARS amounts (μ M) in samples of homogenized fish meat during time and under different storage conditions

**Fig 5:**
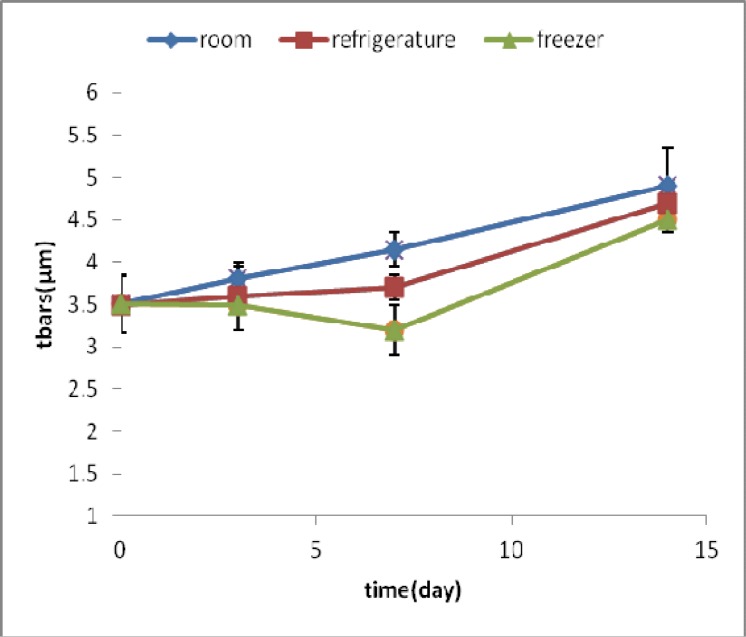
TBARS amount (μ M) in samples of homogenized fish meat containing BHT during time and under different storage conditions

FRAP amount of *Pyrus boissieriana buhse *Leaf extract in zero time was 550±35 μl. The FRAP amounts of test measured during 14 days decreased except on the third day. It means that the FRAP amounts of samples at room, refrigerator and freezer temperatures were decreased about 47%, 20% and 12%, respectively with time from zero to 14 days (Fig. 2). 

TBARS of fish sample containing arbutin in all 3 conditions of room, refrigerator and freezer temperatures decreased till the third day, and then it increased up to the 14th day. 

The most increase at room temperature was about 67%, whereas, the increase in refrigerator and freezer was lower and was about 30% and 11% respectively. (Fig. 3) 

TBARS amount in fish samples containing methanol extract in zero time was 2.4± 0.01 µM which increased about 70% after 14 days. But in samples containing BHT, this amount was 3.01 ±0.13 µM at zero time and increased up to 75% (Figs 4 & 5).

**Fig 6:**
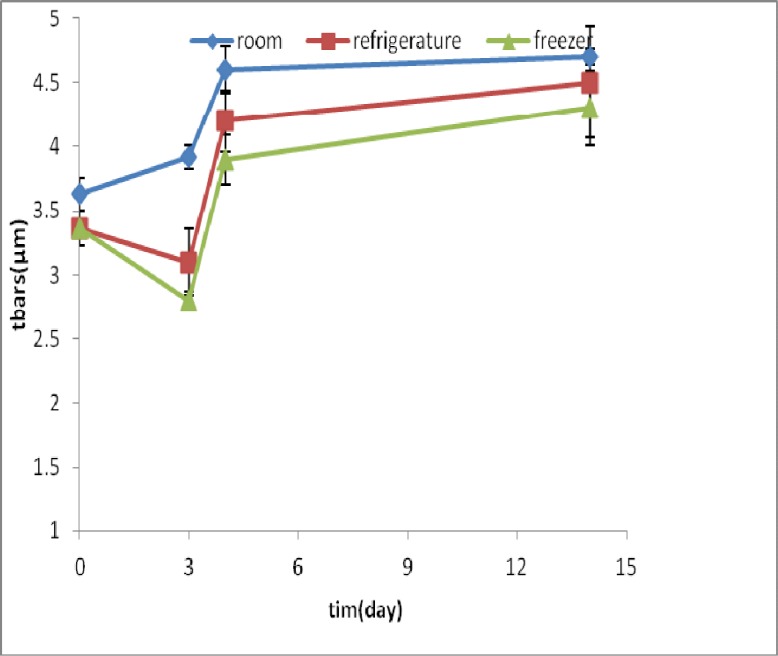
TBARS amount (μM) in samples of homogenized fish meat without extract during time and under different storage conditions

TBARS amount was increased with time in fish samples without extract in all conditions of room, freezer and refrigerator temperatures from 0-14 days. The most increase was at room temperature about 48%, but the increase of TBARS at refrigerator and freezer temperature was 45% and 25%, respectively (Fig. 6).

TBARS amount was increased with time in fish samples without extract in all conditions of room, freezer and refrigerator temperatures from 0-14 days. The most increase was at room temperature about 48%, but the increase of TBARS at refrigerator and freezer temperature was 45% and 25% , respectively (Fig. 6).

**Sustainability of peroxide activities**

In fish samples with arbutin extract, negative and positive controls in all 3 conditions of RT, refrigerator and freezer, during time from 0-14 days, peroxide activities decreased from the beginning. The most decrease was observed at RT temperature during 14 days (Table 1).

**Table1:** peroxidase activities (μM) in samples of homogenized fish meat during time and under different storage conditions

negative control		positive control		extract		arbutin		Conditions		time	
0.4±0.02		0.5±0.04		1±0.03		0.75±0.04		25		3 days	
0.5±0.02		0.8±0.01		1.2±0.07		0.8±0.02		4			
0.7±0.04		0.9±0.05		1.4±0.02		0.9±0.04		-20			
0.2±0.01		0.4±0.02		0.8±0		0.52±0.02		25		7 days	
0.4±0.01		0.6±0.03		0.9±0.04		0.65±0.07		4			
0.6±0.02		0.8±0.03		1.2±0.07		0.79±0.01		-20			
0.1±0.05		0.27±0.01		0.5±0.08		0.35±0		25		14 days	
0.2±0.03		0.5±0.04		0.77±0.02		0.47±0.02		4			
0.4±0.01		0.6±0.03		1±0.06		0.6±0.04		-20			

## Discussion

The content of total phenols with Folin- Ciocalteu method for methanol *Pyrus boissieriana buhse *Leaf extract was 46 mg which was equal to Gallic acid in 1000 ml extract. The amount of flavonoid was equal to 80 mg querceting per g of extract. Phenols and polyphenols like flavonoids are found widely in food production. Flavonoids are important in body health due to their antioxidant activities and their abilities for capturing free radicals. Polyphenols are considered as an important herb compound for their antioxidant properties which have an important role in deleting free radicals and preventing hydroperoxide change into free radicals. The amount of total phenols and flavonoids in this research could satisfy the activities of antioxidants available in plant extract (14). 

FRAP test has shown the existence of antioxidant activities of arbutin and extract and their sustainability within 14 days under different storage conditions. In other studies, decreasing ontioxidant activity were reported with time (15).

The difference in antioxidant capacity of the different extracts in the present study may be due to the presence of the different chemicals including polyphenols, ascorbic acid and carotenoids. Total antioxidant capacity of extract was different from arbutin, because the extract contains arbutin and other active chemical compounds (16). 

The antioxidant, antibacterial, antifungal, antilarval and anticholinesterase activities of a methanolic Leaf extract from this plant have been studied in vitro and the antioxidant, antihyperglycaemic and antihyperlipidemic effects of *Pyrus biossieriana buhse* extract were studied in vivo (6).

In our laboratory, we demonstrated that *Pyrus biossieriana buhse* Leaf extract reduces blood glucose and lipid levels and increases antioxidant status in alloxan-induced hypergly-caemia rats (17).

TBARS test showed that lipid oxidation increased in other conditions, during test times. In the current study, TBARS amounts raised with times in homogenized fish. The most increase was at room temperature and during 14 days. In this research, we demonstrated that the available antioxi-dant in extract cause delay in lipid peroxidation.

In fish homogenate, hydrolysis and oxidation will both make free radicals. Hydrolysis is induced by lipase and phospholipase which release fatty acids which under oxidation, will make low molecular weight compounds. This process is accompanied by free radicals release leading to aldehyde production which is responsible for the spoiled and bad taste and odor and change in meat color (18). 

Lipid oxidation is influenced by internal and external factors including fatty acids, the concen-tration of peroxide, endogen iron Ferrous, myoglo-bin, enzymes, PH, temperature and oxygen usage (19-21). 

Long term TBARS tests showed that higher concentration of extract has more preventive effect. 

Fasseas et al. reported that temperature and storage time decrease antioxidant activity of meat (22). Releasing more free iron and other peroxidants resulted in the increase of lipid oxidation with time. With increasing the time of storage, hydrolysis and lipidoxidation of fish were more and hydroperoxides and conjugated dienes products and reaction of lipid oxidation with TBA increased (22).

The most decrease in peroxidase activity was observed at room temperature with time. 

The study of effect of extract on peroxidase activity showed that extract could decrease the amount of peroxides, which confirms the antioxidant pro-perties of extract and their abilities in free radicals scavengering. So far, there is no study yet similar to our study.

The results of the present study showed the existence of antioxidant activities with high storage stability and lipid oxidation protection effect of arbutin and Leaf extract. Therefore, using arbutin and extract as natural resources of antioxidant is suggested for substituting synthetic antioxidants. It is suggested that using this plant and arbutin with high antioxidant power could be a protective element in several diseases and also it could help adjuvant with new drugs. But it is necessary to investigate the probable side effects for the long term usage of arbutin and* Pyrus boissieriana Buhse *Leaf extract .
